# Neutralization activity of sera/IgG preparations from fully BNT162b2 vaccinated individuals against SARS-CoV-2 Alpha, Beta, Gamma, Delta, and Kappa variants

**DOI:** 10.1038/s41598-022-17071-9

**Published:** 2022-08-08

**Authors:** Masayuki Amano, Sachiko Otsu, Kenji Maeda, Yukari Uemura, Yosuke Shimizu, Kazumi Omata, Masao Matsuoka, Shinya Shimada, Hiroaki Mitsuya

**Affiliations:** 1 Department of Hematology, Rheumatology, and Infectious Diseases, Faculty of Life Sciences, Kumamoto University, Kumamoto, Japan; 2 Department of Refractory Viral Infections, National Center for Global Health and Medicine (NCGM) Research Institute, 1-21-1 Toyama, Shinjuku, Tokyo 162-8655 Japan; 3 Department of Data Sciences, Center for Clinical Sciences, NCGM, Tokyo, Japan; 4 Japan Community Healthcare Organization (JCHO) Kumamoto General Hospital, Kumamoto, Japan; 5 Department of Clinical Sciences, Kumamoto University Hospital, Kumamoto, Japan; 6 Experimental Retrovirology Section, HIV and AIDS Malignancy Branch, National Cancer Institute, National Institutes of Health, Bethesda, MD USA

**Keywords:** Viral infection, Outcomes research

## Abstract

In the present prospective study, 225 individuals in Kumamoto General Hospital, Japan, who received two-doses of BNT162b2 vaccine were enrolled/followed up over 150 days and neutralizing activity (NT_50_) of their sera and antiviral activity (EC_50_) of IgG purified from sera on day-60 post-1st-dose were determined against wild-type SARS-CoV-2 (SARS-CoV-2^Wuhan^) (n = 211) and 9 variants (Alpha, Beta, Gamma, Delta, and Kappa) (n = 45). Time-dependent changes of IgG-activity (n = 25) against SARS-CoV-2^Wuhan^ and variants were also examined. Day-60 sera showed reduced NT_50_ by more than 50% against all variants examined, and greatest reduction was seen with Beta. IgG fractions of high-responders and moderate-responders showed similar fold-changes in EC_50_ against each variant compared to SARS-CoV-2^Wuhan^. Evaluation of EC_50_ of IgG obtained at different time-points (day-28 to -150) revealed time-dependent reduction of activity against all variants. However, against Delta, relatively long-lasting favorable antiviral activity (at least 150 days) was observed. Our data strongly suggest that the successful antecedent scale-up of mRNA-based vaccine administrations in Japan was the primary contributor to the lessening of the otherwise more devastating SARS-CoV-2 pandemic wave caused by the Delta variant. The present data that the effectiveness of vaccine against the then-dominant SARS-CoV-2 variant was likely associated with the moderation of the COVID-19 pandemic wave suggest that as in the case of influenza vaccines, the development of multivalent mRNA-based vaccines represent a generalizable approach to pre-emptively respond pandemic with mutable pathogens.

## Introduction

Since the emergence of coronavirus disease 2019 (COVID-19) caused by the severe acute respiratory syndrome coronavirus 2 (SARS-CoV-2) infection in Wuhan, COVID-19 rapidly spread to the world as never before. Globally, 424.8 million SARS-CoV-2-confirmed cases and more than 5.8 million of death cases by COVID-19 have been reported as of February 22, 2022^1–4^. From the initial stage of the global pandemic, researchers and pharmaceutical companies around the world have been making massive efforts toward development of novel vaccines against SARS-CoV-2^5^. To date, more than 30 vaccines have been approved by at least one country (https://covid19.trackvaccines.org/vaccines/approved/). The efficacy of vaccines against SARS-CoV-2 has been beyond expectations, as of February 22, more than 10.4 billion of vaccine doses have already been administered in the world (https://covid19.who.int/).

Among various vaccines, two mRNA vaccines BNT162b2 (Pfizer/BioNTech) and mRNA-1273 (Moderna), have shown 94–95% efficacy in preventing symptomatic COVID-19^6–8^. Additionally, inactivated vaccines or viral vector vaccines have also been used in certain countries^5,7–9^. For instance, the adenovirus vector-based vaccine, AZD1222 (ChAdOx1 nCoV-19, Oxford/AstraZeneca), has reportedly showed 62.1% efficacy in initial clinical trials^10^. More recent study reported that robust immune responses accompanied by acceptable reactogenicity after heterologous vaccination of AZD1222 prime and BNT162b2 or mRNA-1273 boost^11,12^. Another adenovirus-based vaccine, Ad26.COV2.S (Janssen), has shown 85.4% prevention efficacy against severe/critical COVID-19^13,14^.

However, the recent emergence of various SARS-CoV-2 variants with mutations in the spike-encoding region is raising global concerns about the efficacy of vaccines against such variants. The D614G and B.1.1.7 (Alpha/N501Y) variants might have improved viral fitness, but apparently not immune escape^15,16^. However, the B.1.351 (Beta) variant, containing E484K substitutions, is reportedly represents a neutralization escape variant to convalescent sera^17^. A phase 3 trial of NVX-CoV2373 (Novavax), a recombinant protein-based nanoparticle vaccine indicated 86.3% efficacy to the more transmissible B1.1.7 (Alpha) variant in a post hoc analysis^18,19^. However, a phase 2b trial in South Africa showed 50.1% vaccine efficacy against the B1.351 (Beta) variant in a post hoc analysis^20^, suggesting that the Beta variant is less susceptible to antibodies elicited with the original Wuhan strain’s antigens, which is in the composition of all the vaccines currently being evaluated^9^. P.1 (Gamma) variants also reported to evade antibody responses induced by previous infection or vaccination^21^.

Another recent concern is the emergence of a B.1.617.2 (Delta) variant, which was first detected in India, has been spreading around the world. This variant of concern (VOC) has reportedly less susceptibility to vaccine-elicited protection and increased transmissibility beyond Alpha strains^22^. B1.617.1 (Kappa) variant, which belongs to the same lineage with the Delta variant and WHO classified as variant under monitoring (VUM), has characteristic E484Q mutation along with L452R and D614G mutations in the spike-encoding region, reportedly have reduced susceptibility to neutralization activity of therapeutic monoclonal antibodies and convalescent/vaccinated sera, in experiments using pseudoviruses^23^.

In the present study, we examined neutralizing activity of sera obtained at day-60 post 1st dose from BNT162b2-vaccinated health care workers in Japan, against wild-type Wuhan strain (n = 211) and 9 distinct SARS-CoV-2 variants, including various VOCs (Alpha, Beta, Gamma, and Delta) and VUM (Kappa) (n = 45). All the SARS-CoV-2 strain/variants used in this study were infectious viruses, neither recombinant-virus nor pseudo-virus, and isolated from individuals at airport quarantine station or hospital in Japan. We also investigated antiviral activity of IgG obtained from day-60 sera of 45 participants (classified as group of high responders and group of moderate responders, by the results of neutralizing activity of each serum against wild-type Wuhan strain) against wild-type and various variants. Finally, we eluted IgG fractions from sera of 25 participants at day-28, -90, and -150, then examined time-dependent alternations of antiviral activity of each IgG against Wuhan strain, VOCs, and VUM.

## Results

### Immune response in the participants on day-60 post 1st dose

We obtained blood samples for neutralizing activity evaluation from a total of 225, 220, 211, 210, and 208 vaccine recipients on days 7, 28, 60, 90, and 150 post 1st doses, respectively^24^. Demographic characteristics of the participants are shown in Table 1. As of the time of enrollment, the average age of the participants was 41.8 years (range 21 to 72 years), and 69.8% of the participants were female serving as a physician, nurse, paramedical staff, or administrative staff. None of the participants was in the immunodeficient state or was receiving immunosuppressants or steroids.

**Table 1:** Demographic characteristics of the participants.

	Day-7	Day-28	Day-60	Day-90	Day-150
	(1 week post 1st dose)	(1 week post 2nd dose)			
Participants (all)*	225	220	211	210	208
**Age**					
20–39 y.o.	97 (43.1%)	92	84	84	82
40–59 y.o.	110 (48.9%)	110	109	108	109
60–y.o.	18 (8.0%)	18	18	18	17
**Gender**					
Men	68 (30.2%)	68	63	61	62
Women	157 (69.8%)	152	148	149	146
**Job**					
Physicians	36 (16.0%)				
Nurses	125 (55.6%)				
Others	64 (28.4%)				

*None of the participants were in immunodeficient states or were receiving immunosuppressants or steroids. Two participants had previously experienced SARS-CoV-2 infection and were excluded from the present study since day-28. None of other participants experienced COVID-19-related symptoms and proved to be negative for antibodies against serum SARS-CoV-2 nucleocapsid throughout the study period.

**Figure 1:**
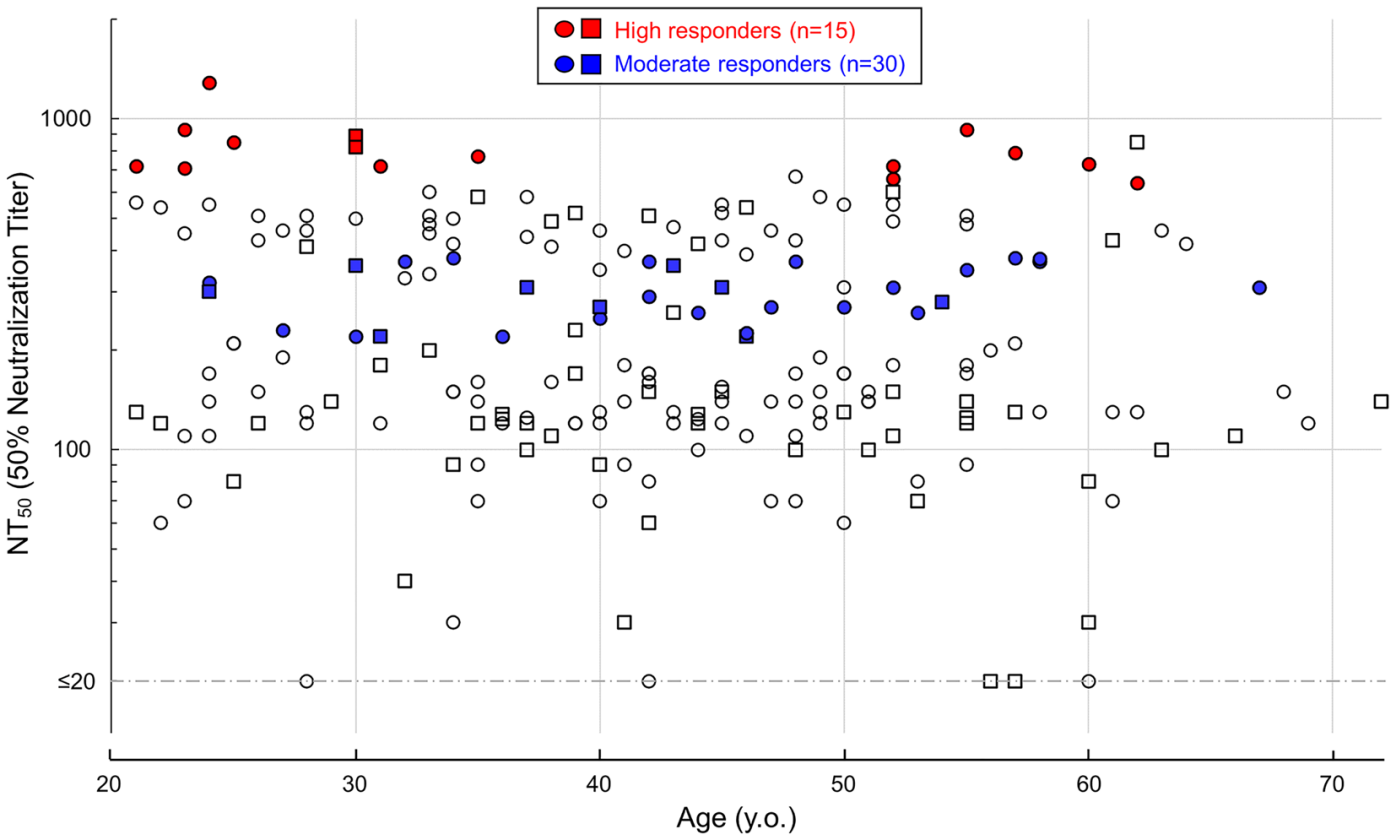
Neutralization of wild-type SARS-CoV-2^05-2N^ by day-60 sera. Neutralization activity of day-60 sera obtained from participant were shown. Day-60 participants whose sera showed NT_50_ values against SARS-CoV-2^05-2N^ greater than 500-fold were classified as high responders (open red circles, n = 15), and participants whose sera showed NT_50_ values around average of all participants (range 220 to 380-fold) were categorized as moderate responders (open blue circles, n = 30).

In the present study, we focused upon the blood samples taken on day-60 post 1st dose from 211 vaccinated participants. First, we determined the neutralizing activity of each serum against cytopathic effect induced by wild-type SARS-CoV-2^05-2N^ infection and replication in the VeroE6 cell-based assay. As shown in Fig. 1, 50% neutralization titer (NT_50_) levels were substantially diverse among the participants: the average NT_50_ value was 284 (range 30–1290), and five participant’s sera showed NT_50_ values less than cut-off level (< 20).

### High and moderate responder’s sera show reduced anti-SARS-CoV-2 activity against various VOCs and VUM

Next, we selected and designated day-60 participants, whose sera had NT_50_ values against SARS-CoV-2^05-2N^ greater than 500 as high responders (n = 15; shown in red), and participants, whose sera had NT_50_ values around average of all participants (range 220 to 380) as moderate responders (n = 30; shown in blue) (Fig. 1), and evaluated neutralizing activity of each serum belonging above mentioned two groups against nine infectious SARS-CoV-2 variants; variants of concerns (VOCs) and variants under monitoring (VUM), including Alpha, Beta, Gamma, Delta, Kappa, and E484K/D614G- amino acid subs carrying variants (E484K variants).

**Figure 2:**
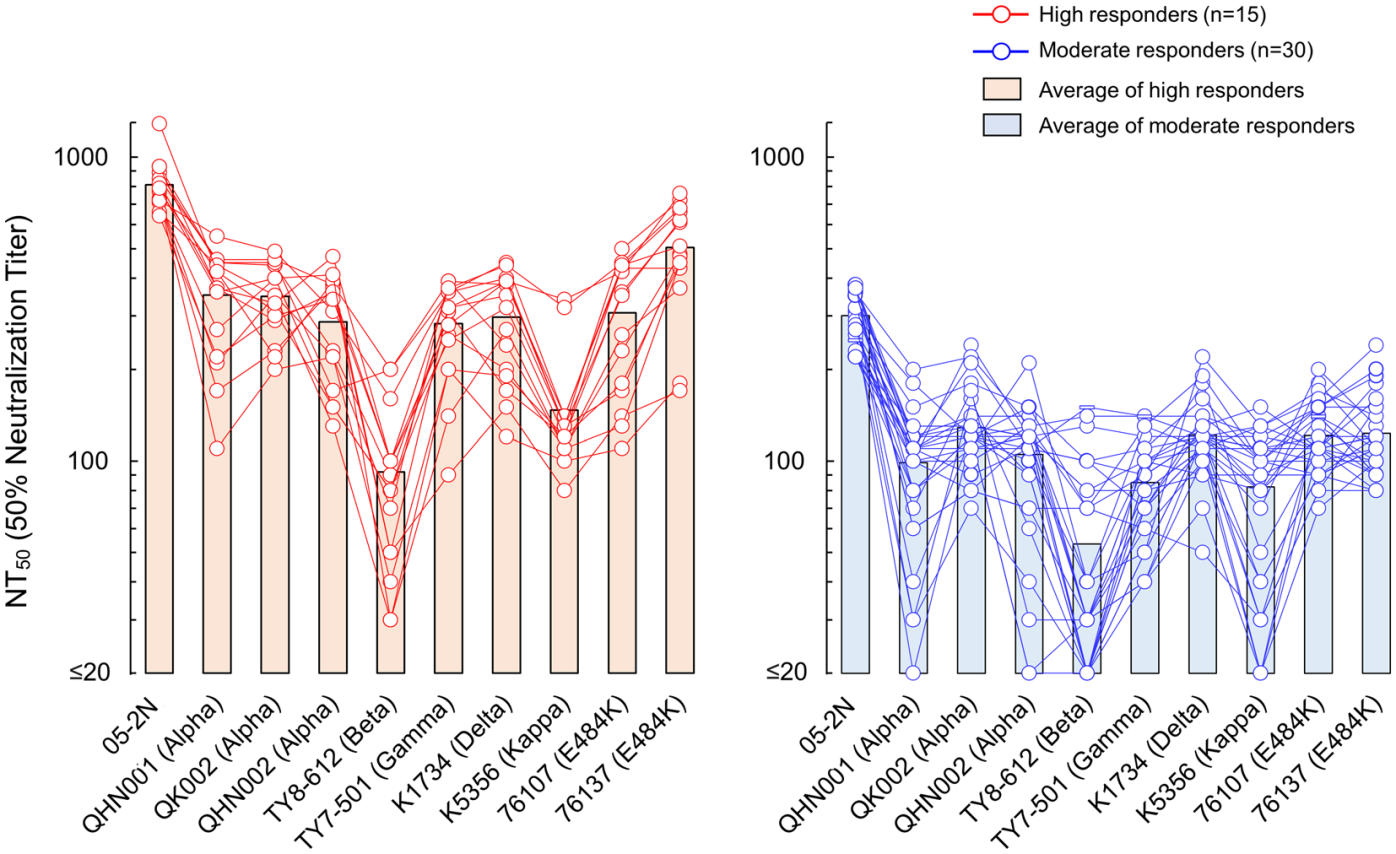
Neutralization of VOCs and VUM by day-60 sera from high and moderate responders. The activity of vaccinees’ sera to block the infectivity and replication of 9 SARS-CoV-2 variants (Alpha variants: SARS-CoV-2^QHN001^, SARS-CoV-2^QK002^, and SARS-CoV-2^QHN002^; a Beta variant: SARS-CoV-2^TY8-612^; a Gamma variant: SARS-CoV-2^TY7-501^; a Delta variant: SARS-CoV-2^K1734^; a Kappa variant: SARS-CoV-2^K5356^; and E484K variants: SARS-CoV-2^76107^ and SARS-CoV-2^76137^) were evaluated. A Wuhan strain SARS-CoV-2^05-2N^ was employed as a reference SARS-CoV-2. Left panel shows results of high responders (n = 15), whereas right panel shows the results of moderate responders (n = 30). The NT_50_ titers of each serum against SARS-CoV-2 variant are shown in red (high responders) or blue (moderate responders) circles, and bar represent average NT_50_ titer of each group. *P* values for the difference between SARS-CoV-2^05-2N^ and each variant of high responders or moderate responders: < 0.001 (QHN001), < 0.001 (QK002), < 0.001 (QHN002), < 0.001 (TY8-612), < 0.001 (TY7-501), < 0.001 (K1734), < 0.001 (K5356), < 0.001 (76107), and < 0.001 (76137).

Average NT_50_ values of high responder’s sera against three Alpha variants, SARS-CoV-2^QHN001^, SARS-CoV-2^QK002^, and SARS-CoV-2^QHN002^ were 351, 348, and 287, respectively, and %reductions from the average NT_50_ value against SARS-CoV-2^05-2N^ (811) were by 56.7, 57.1, and 64.6%, respectively (Fig. 2, Table 2). High responder’s average NT_50_ value against the Beta variant, SARS-CoV-2^TY8-612^, decreased remarkably to 92 (%reduction by 88.7%). High responder’s sera showed moderate reduction with the Gamma variant, SARS-CoV-2^TY7-501^ with their average NT_50_ value being 283 and %reduction by 65.1% (Fig. 2, Table 2). Against the Kappa variant, SARS-CoV-2^K5356^, high responder’s sera showed high reduction level of neutralizing activity, with NT_50_ value being 147 and %reduction by 81.9%, whereas high responder’s sera showed lesser reduction against the Delta variant, SARS-CoV-2^K1734^ with NT_50_ of 297 and %reduction by 63.4% (Fig. 2, Table 2). We also examined neutralizing activity of sera against two E484K-carrying variants, SARS-CoV-2^F-76107^ and SARS-CoV-2^F-76137^, which were isolated in Japan before the emergence of Alpha variants. High responder’s sera showed average NT_50_ value of 307 to 504 against both variants with reduction by 37.9–62.1% (Fig. 2, Table 2). As for the group of moderate responders, similar trends were observed with the results of high responder’s group, neutralizing activity more significantly reduced against Beta, Kappa, and Gamma variants, with reduction by 83.4, 73.4, and 71.8%, respectively. Against the other strains tested, moderate responder’s sera showed reduction by 57.5 to 67.4% (Fig. 2, Table 2).

**Table 2:** Reduction of neutralization against VOCs and VUM by day-60 sera from high and moderate responders compared to those against wild-type Wuhan strain.

Strains	Wuhan	Alpha			Beta	Gamma	Delta	Kappa	E484K	
	05-2N	QHN001	QK002	QHN002	TY8-612	TY7-501	K1734	K5356	F-76107	F-76137
**(A) High responder’s sera (n = 15)**										
Average NT_50_	811 ± 155	351 ± 122	348 ± 88	287 ± 107	92 ± 53	283 ± 89	297 ± 108	147 ± 74	307 ± 133	504 ± 171
*Reduction (%)	–	56.7	57.1	64.6	88.7	65.1	63.4	81.9	62.1	37.9
*p* value vs 05-2N	–	< 0.001	< 0.001	< 0.001	< 0.001	< 0.001	< 0.001	< 0.001	< 0.001	< 0.001
**(B) Moderate responder’s sera (n = 30)**										
Average NT_50_	301 ± 56	98 ± 40	128 ± 40	105 ± 41	53 ± 39	85 ± 29	122 ± 32	82 ± 38	121 ± 31	123 ± 38
*Reduction (%)	–	67.4	57.5	65.4	83.4	71.8	59.5	73.4	59.8	59.1
*p* value vs 05-2N	–	< 0.001	< 0.001	< 0.001	< 0.001	< 0.001	< 0.001	< 0.001	< 0.001	< 0.001

Average NT_50_ titers of day-60 sera of high (n = 15) or moderate (n = 30) responders against SARS-CoV-2^05-2N^ and various variants are indicated.

*%Reduction of NT_50_ titers compared to that against SARS-CoV-2^05-2N^.

Overall, sera in both groups showed reduced neutralizing activity by greater than 50% against all variants examined in this study compared to those against wild-type SARS-CoV-2^05-2N^, and the greatest reduction of neutralizing activity occurred with the Beta variant.

### IgG fractions obtained from high and moderate responders’ sera show different properties regarding anti-SARS-CoV-2 activity against various VOCs and VUM

In attempt to further quantify anti-SARS-CoV-2 activity induced by BNT162b2, we obtained IgG fractions from each of high and moderate responder’s serum and carried out the VeroE6^TMPRSS2^ cell-based anti-SARS-CoV-2 assay determining EC_50_ (50% effective concentration: μg/ml) value of each IgG fraction against wild-type SARS-CoV-2^05-2N^ and nine distinct SARS-CoV-2 variants. We believe that such attempt can exclude any effects caused by non-IgG factors existing in sera, enable to evaluate actual neutralizing activity of IgG alone as induced by the BNT162b2 vaccination against SARS-CoV-2.

**Figure 3:**
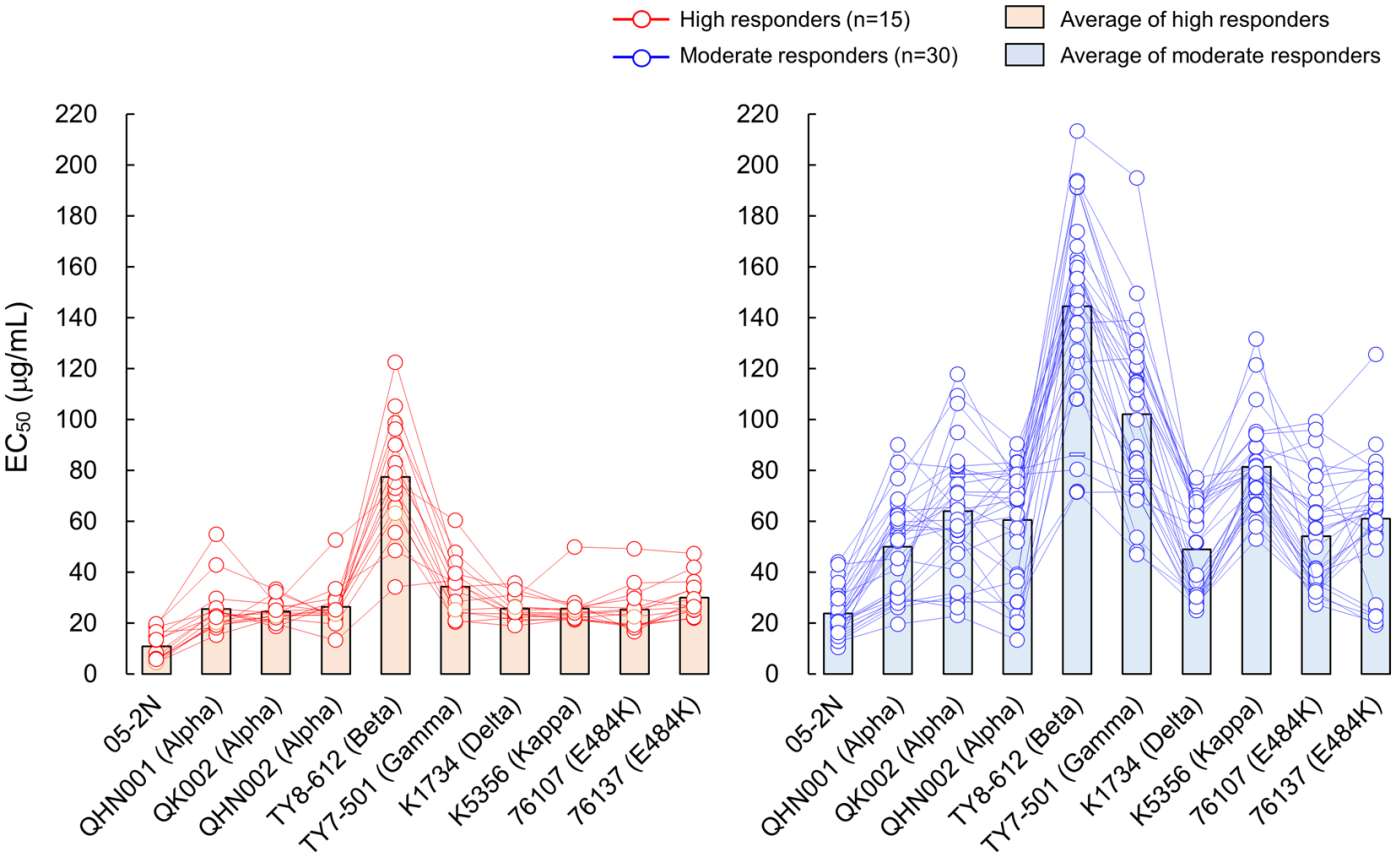
Antiviral activity of IgG fractions eluted from day-60 sera of high and moderate responders against VOCs and VUM. The antiviral activity of IgG fractions eluted from vaccinees’ sera against 9 SARS-CoV-2 variants was evaluated. A Wuhan strain SARS-CoV-2^05-2N^ was employed as a reference SARS-CoV-2. Left panel shows results of high responders (n = 15), whereas right panel shows the results of moderate responders (n = 30). The EC_50_ values of each IgG against SARS-CoV-2 variant are shown in red (high responders) or blue (moderate responders) circles, and bar represent average EC_50_ value of each group. *P* values for the difference between SARS-CoV-2^05-2N^ and each variant of high responders or moderate responders: < 0.001 (QHN001), < 0.001 (QK002), < 0.001 (QHN002), < 0.001 (TY8-612), < 0.001 (TY7-501), < 0.001 (K1734), < 0.001 (K5356), < 0.001 (76107), and < 0.001 (76137).

As shown in Fig. 3 and Table 3, average EC_50_ values of IgG eluted from high responder’s sera and moderate responder’s sera against SARS-CoV-2^05-2N^ were 10.8 and 23.8 μg/ml, respectively. Against three Alpha variants, SARS-CoV-2^QHN001^, SARS-CoV-2^QK002^, and SARS-CoV-2^QHN002^, high responder’s IgG (IgG^high^s) showed EC_50_ values of 25.6, 24.5, and 26.3 μg/ml, respectively, and fold changes compared with EC_50_ value against SARS-CoV-2^05-2N^ were 2.4, 2.3, and 2.4, respectively. On the other hand, moderate responder’s IgG (IgG^moderate^s) generally showed more than two-times higher EC_50_ values compared to those of IgG^high^s, as 50.1, 64.0, and 61.4 μg/ml, respectively, against the same three Alpha variants, and fold changes were by 2.1, 2.7, and 2.6, respectively.

**Table 3:** Anti-SARS-CoV-2 activity and fold changes of IgG fractions eluted from day-60 sera against VOCs and VUM compared to those against wild-type Wuhan strain.

Strains	Wuhan	Alpha			Beta	Gamma	Delta	Kappa	E484K	
	05-2N	QHN001	QK002	QHN002	TY8-612	TY7-501	K1734	K5356	F-76107	F-76137
**(A) High responder’s sera (n = 15)**										
Average EC_50_ (μg/ml)	10.8 ± 5.6	25.6 ± 10.0	24.5 ± 4.0	26.3 ± 8.3	77.3 ± 22.2	34.2 ± 10.7	25.7 ± 4.6	25.7 ± 6.8	25.3 ± 8.3	30.1 ± 7.0
Fold changes*	–	2.4	2.3	2.4	7.2	3.2	2.4	2.4	2.3	2.8
*p* value vs 05-2N	–	< 0.001	< 0.001	< 0.001	< 0.001	< 0.001	< 0.001	< 0.001	< 0.001	< 0.001
**(B) Moderate responder’s sera (n = 30)**										
Average EC_50_ (μg/ml)	23.8 ± 8.8	50.1 ± 18.2	64.0 ± 24.0	60.5 ± 22.2	144.5 ± 35.9	102.1 ± 32.7	49.0 ± 17.9	81.3 ± 16.8	54.1 ± 21.1	61.0 ± 23.6
Fold changes*	–	2.1	2.7	2.5	6.1	4.3	2.1	3.4	2.3	2.6
*p* value vs 05-2N	–	< 0.001	< 0.001	< 0.001	< 0.001	< 0.001	< 0.001	< 0.001	< 0.001	< 0.001

Average EC_50_ values of IgG fraction eluted from day-60 sera of high (n = 15) or moderate (n = 30) responders against SARS-CoV-2^05-2N^ and various variants are indicated.

*Fold change of EC_50_ values compared to that against SARS-CoV-2^05-2N^.

High responder’s average EC_50_ value against the Beta variant, SARS-CoV-2^TY8-612^, was as high as 77.3 μg/ml, the greatest value among variants we tested, and fold change was by 7.2. IgG^high^s showed moderate reduction of antiviral activity against Gamma variant, SARS-CoV-2^TY7-501^, their average EC_50_ and fold change were 34.2 μg/ml and 3.2-fold, respectively (Fig. 3, Table 3). Against Delta and Kappa variants, SARS-CoV-2^K1734^ and SARS-CoV-2^K5356^, IgG^high^s showed same EC_50_ value, 25.7 μg/ml, and fold changes were similar with those against Alpha variants (Fig. 3, Table 3). Also, antiviral activity of IgG^high^s against two E484K variants, SARS-CoV-2^F-76107^ and SARS-CoV-2^F-76137^, were similar level with Alpha variants, average EC_50_ values were 25.3 and 30.1 μg/ml, respectively, and fold changes were 2.3 and 2.8, respectively (Fig. 3, Table 3).

On the other hand, the average EC_50_ value of IgG^moderate^s against SARS-CoV-2^05-2N^ was 23.8 μg/ml, 2.2-fold higher than that of IgG^high^s. Also, average EC_50_ values of IgG^moderate^s against various VOCs and VUM we tested were higher than those of IgG^high^s with range of 1.9–3.2-folds (49.0–144.5 μg/ml; Fig. 3, Table 3).

We also carried out comparison between NT_50_ values (sera) and EC_50_ values (IgG preparations) of randomly selected participants [the high (n = 15) and moderate (n = 30) responders: a total of 45 participants] using data obtained various strains. As shown in Fig. S1, significant inverse correlations between the two values against the ancestral Wuhan strain and 4 VOCs (Alpha, Beta, Gamma, and Delta variants) were observed (Spearman ρ values =  − 0.82 to − 0.59; p < 0.0001).

### Evaluation of anti-SARS-CoV-2 activity of IgG fractions obtained at different time points reveals relatively long-lasting antiviral activity of IgG against Delta-variant

Next, we also eluted IgG fractions from sera of 25 participants at day-28 and day-90 (n = 13 for IgG^high^s and n = 12 for IgG^moderate^s), then determined EC_50_ values of each IgG fraction against SARS-CoV-2^05-2N^, various VOCs, and VUM.

**Figure 4:**
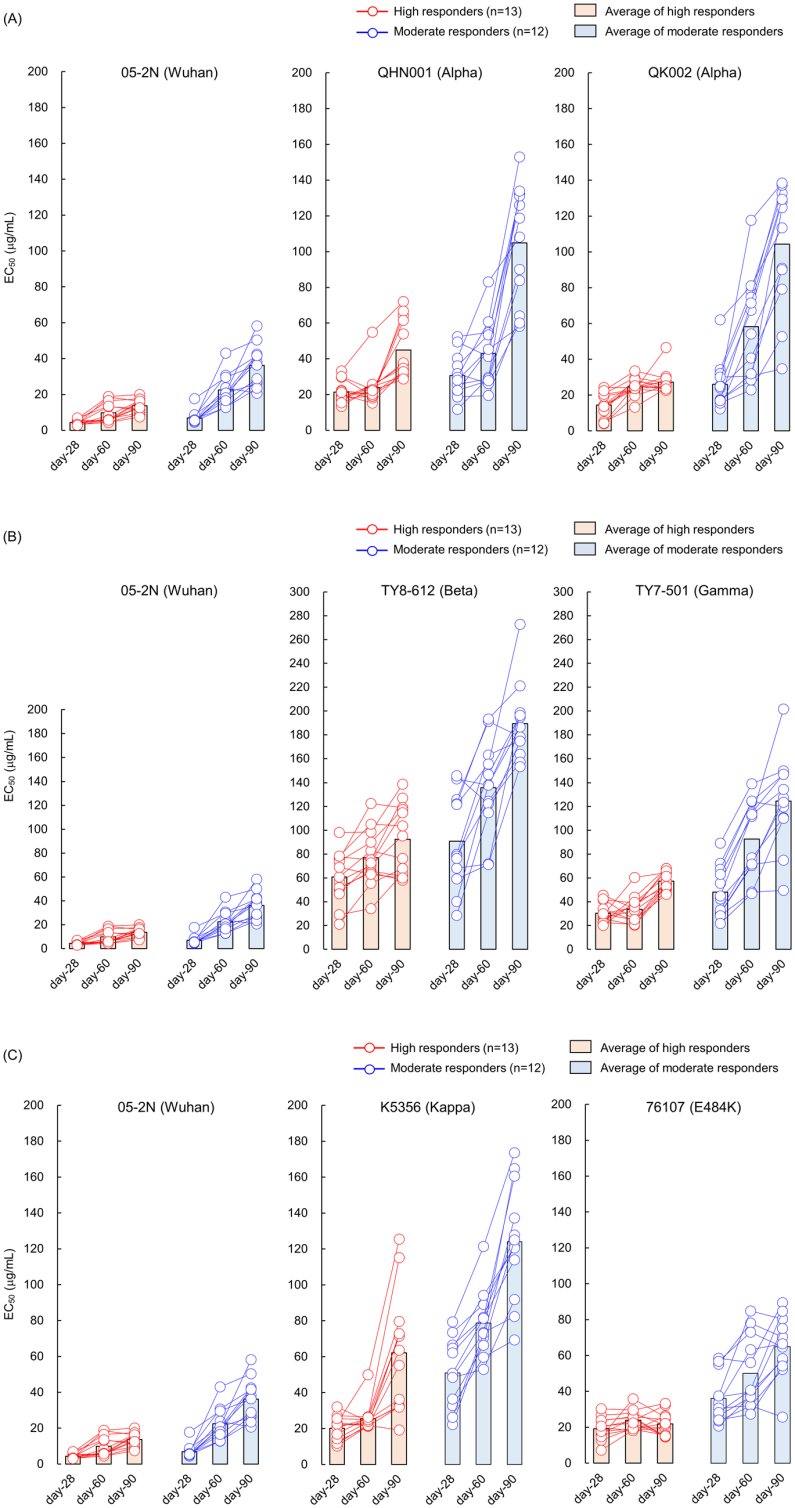
Anti-SARS-CoV-2 activity of IgG fractions eluted from day-28, -60, and -90 sera of high and moderate responders against VOCs and VUM. Time dependent alterations of antiviral activity of IgG fractions eluted from day-28, -60, and -90 sera against 7 SARS-CoV-2 variants (Alpha variants: SARS-CoV-2^QHN001^ and SARS-CoV-2^QK002^; a Beta variant: SARS-CoV-2^TY8-612^; a Gamma variant: SARS-CoV-2^TY7-501^; a Delta variant: SARS-CoV-2^K1734^; a Kappa variant: SARS-CoV-2^K5356^; and a E484K variant: SARS-CoV-2^76107^). A Wuhan strain SARS-CoV-2^05-2N^ was employed as a reference SARS-CoV-2. (**A**) Shows the EC_50_ results against 2 Alpha variants, (**B,C**) show the EC_50_ results against Beta/Gamma variants, and Kappa/E484K variants, respectively. Each panel contains the EC_50_ results against SARS-CoV-2^05-2N^ for comparison. The EC_50_ values of each IgG against SARS-CoV-2 variant are shown in red (high responders; n = 13) or blue (moderate responders; n = 12) circles, and bar represent average EC_50_ value of each group.

As shown in Fig. 4 and Table 4, both IgG groups maintained good anti-SARS-CoV-2 activity against SARS-CoV-2^05-2N^, average EC_50_ values of IgG^high^s and IgG^moderate^s from day-28 sera were 4.3 and 6.9 μg/ml, respectively, and 13.7 and 36.2 μg/ml, respectively, for the IgG eluted from day-90 sera.

**Table 4:** Anti-SARS-CoV-2 activity of IgG fractions eluted from day-28, 60, 90, and 150 sera of high and moderate responders against VOCs and VUM.

Strain/variant		Wuhan/SARS-CoV-2^05-2N^				Alpha/SARS-CoV-2^QHN-001^			
Days from 1st dose		Day-28	Day-60	Day-90		Day-28	Day-60	Day-90	
**(A)**									
Average EC_50_ (μg/ml)	High responder’s IgG	4.3 ± 1.3	9.8 ± 5.3	13.7 ± 3.6		21.4 ± 5.9	23.9 ± 9.4	44.9 ± 15.4	
	Moderate responder’s IgG	6.9 ± 3.5	22.5 ± 8.2	36.2 ± 10.9		30.7 ± 11.7	43.0 ± 17.4	104.9 ± 31.3	
Fold changes*	High responder’s IgG	–	2.3	3.2		–	1.1	2.1	
	Moderate responder’s IgG	–	3.3	5.3		–	1.4	3.4	
Moderate/high ratio		1.6	2.3	2.7		1.4	1.8	2.3	
Mean of fold change**	High responder’s IgG	–	2.3	3.4		–	1.2	2.1	
	Moderate responder’s IgG	–	3.6	5.8		–	1.5	3.8	
	Difference (95% CI)	–	− 1.25 (− 2.43, − 0.07)	− 2.44 (− 3.76, − 1.12)		–	− 0.33 (− 0.73, 0.07)	− 1.68 (− 2.65, − 0.71)	
	*p* value	–	0.039	0.001		–	0.1	0.002	

Variant		Alpha/SARS-CoV-2^QK002^				Beta/SARS-CoV-2^TY8-612^			
Days from 1st dose		Day-28	Day-60	Day-90		Day-28	Day-60	Day-90	
Average EC_50_ (μg/ml)	High responder’s IgG	14.4 ± 6.6	24.7 ± 6.1	27.2 ± 6.1		60.8 ± 20.4	77.0 ± 22.1	92.6 ± 28.0	
	Moderate responder’s IgG	26.0 ± 12.8	58.3 ± 27.2	104.3 ± 33.2		90.8 ± 38.1	135.9 ± 37.3	189.3 ± 31.2	
Fold changes*	High responder’s IgG	–	1.7	1.9		–	1.3	1.5	
	Moderate responder’s IgG	–	2.2	4		–	1.5	2.1	
Moderate/high ratio		1.8	2.4	3.8		1.5	1.8	2	
Mean of Fold change**	High responder’s IgG	–	2.3	2.5		–	1.4	1.7	
	Moderate responder’s IgG	–	2.5	4.7		–	1.8	2.6	
	Difference (95% CI)	–	− 0.21 (− 1.36 , 0.94)	− 2.11 (− 3.7, − 0.53)		–	− 0.44 (− 1.06, 0.18)	− 0.94 (− 1.87, − 0.02)	
	*p* value	–	0.708	0.011		–	0.159	0.047	

Variant		Gamma/SARS-CoV-2^TY7-501^				Delta/SARS-CoV-2^K1734^			
Days from 1st dose		Day-28	Day-60	Day-90		Day-28	Day-60	Day-90	
Average EC_50_ (μg/ml)	High responder’s IgG	30.3 ± 7.8	33.6 ± 10.7	57.4 ± 7.1		10.3 ± 5.6	25.2 ± 4.7	22.0 ± 5.6	
	Moderate responder’s IgG	48.2 ± 20.2	92.7 ± 30.5	124.6 ± 36.8		31.8 ± 12.6	46.7 ± 19.9	65.9 ± 23.1	
*Fold changes	High responder’s IgG	–	1.1	1.9		–	2.5	2.1	
	Moderate responder’s IgG	–	1.9	2.6		–	1.5	2.1	
Moderate/high ratio		1.6	2.8	2.2		3.1	1.9	3.0	
Mean of fold change	High responder’s IgG	–	1.2	2		–	3.4	3.3	
	Moderate responder’s IgG	–	2	2.8		–	1.5	2.2	
	Difference (95% CI)	–	− 0.82 (− 1.18, − 0.46)	− 0.83 (− 1.51, − 0.14)		–	1.95 (0.65, 3.25)	1.02 (− 0.57, 2.62)	
	*p* value	–	< 0.001	0.02		–	0.005	0.198	

Variant		Kappa/SARS-CoV-2^K5356^				E484K/SARS-CoV-2^F-76107^			
Days from 1st dose		Day-28	Day-60	Day-90		Day-28	Day-60	Day-90	
Average EC_50_ (μg/ml)	High responder’s IgG	20.1 ± 6.3	25.6 ± 7.2	62.1 ± 31.2		19.2 ± 5.9	23.9 ± 5.0	21.9 ± 6.1	
	Moderate responder’s IgG	51.0 ± 17.9	78.8 ± 17.1	124.1 ± 31.0		36.0 ± 13.0	50.1 ± 19.2	64.9 ± 16.5	
Fold changes*	High responder’s IgG	–	1.3	3.1		–	1.2	1.1	
	Moderate responder’s IgG	–	1.5	2.4		–	1.4	1.8	
Moderate/high ratio		2.5	3.1	2		1.9	2.1	3	
Mean of fold change**	High responder’s IgG	–	1.4	3.4		–	1.4	1.3	
	Moderate responder’s IgG	–	1.7	2.8		–	1.4	2	
	Difference (95% CI)	–	− 0.33 (− 0.8, 0.14)	0.61 (− 0.9, 2.11)		–	− 0.06 (− 0.45, 0.33)	− 0.71 (− 1.23, − 0.19)	
	*p* value	–	0.157	0.412		–	0.757	0.01	

Strain/variant		Wuhan/SARS-CoV-2^05-2N^				Delta/SARS-CoV-2^K1734^			
Days from 1st dose		Day-28	Day-60	Day-90	Day-150	Day-28	Day-60	Day-90	Day-150
**(B)**									
Average EC_50_ (μg/ml)	High responder’s IgG	4.3 ± 1.3	9.8 ± 5.3	13.7 ± 3.6	21.8 ± 5.5	10.3 ± 5.6	25.2 ± 4.7	22.0 ± 5.6	30.1 ± 6.4
	Moderate responder’s IgG	6.9 ± 3.5	22.5 ± 8.2	36.2 ± 10.9	55.4 ± 18.5	31.8 ± 12.6	46.7 ± 19.9	65.9 ± 23.1	77.8 ± 11.0
Fold changes*	High responder’s IgG	–	2.3	3.2	5.1	–	2.5	2.1	2.9
	Moderate responder’s IgG	–	3.3	5.3	8.0	–	1.5	2.1	2.4
Moderate/high ratio		1.6	2.3	2.7	2.5	3.1	1.9	3.0	2.6
Mean of fold change**	High responder’s IgG	–	2.3	3.4	5.4	–	3.4	3.3	4.3
	Moderate responder’s IgG	–	3.6	5.8	9.0	–	1.5	2.2	2.8
	Difference (95% CI)	–	− 1.25 (− 2.43, − 0.07)	− 2.44 (− 3.76, − 1.12)	− 3.60 (− 6.25, − 0.96)	–	1.95 (0.65, 3.25)	1.02 (− 0.57, 2.62)	1.53 (− 0.45, 3.51)
	*p* value	–	0.039	0.001	0.01	–	0.005	0.198	0.123

Average EC_50_ values of IgG fraction eluted from sera of high (n = 13) or moderate (n = 12) responders, taken on day-28, -60, -90, and -150 post 1st vaccine doses, are indicated.

*Fold change of EC_50_ values compared to that of day-28 IgG.

**Mean of fold change was calculated by arithmetic mean of each participant’s fold change at day-60, -90, and -150 against day-28. The difference between high and moderate responders using Student’s t-test.

Against Alpha variants (QHN001 and QK002), IgG from day-28 sera of both groups relatively maintain their anti-SARS-CoV-2 activity, average EC_50_ values of IgG^high^s and IgG^moderate^s were lower than 50 μg/ml (21.4–30.7 μg/ml for QHN001 and 14.4–26.0 μg/ml for QK002), and EC_50_ values were gradually increased time-dependently, IgG^high^s from day-90 sera showed EC_50_ values of 44.9 and 27.2 μg/ml against QHN001 and QK002, respectively, and day-90 IgG^moderate^s showed EC_50_ values of 104.9 and 104.3 μg/ml, respectively (Fig. 4A, Table 4A).

IgG from day-28 sera of both groups showed reduced anti-SARS-CoV-2 activity against Beta variant, average EC_50_ values of IgG^high^s and IgG^moderate^s were 60.8 and 90.8 μg/ml, respectively, and values continued to increase up to 92.6 and 189.3 μg/ml, respectively, for IgG from day-90 sera (Fig. 4B, Table 4A).

Against Gamma variant, IgG from day-28 sera of both groups relatively maintained anti-SARS-CoV-2 activity with EC_50_ values lower than 50 μg/ml (30.3 μg/ml for IgG^high^s and 48.2 μg/ml for IgG^moderate^s), although values elevated up to 57.4 and 124.6 μg/ml for day-90 IgG^high^s and IgG^moderate^s, respectively (Fig. 4B, Table 4A).

**Figure 5:**
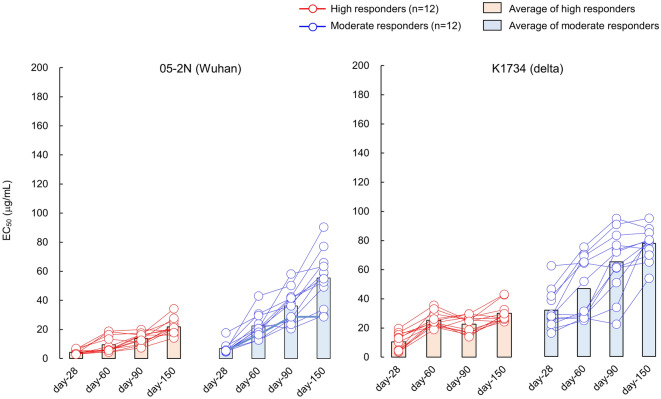
Anti-SARS-CoV-2 activity of IgG fractions eluted from day-28, -60, -90, and -150 sera of high and moderate responders against Delta variant. Time dependent alterations of antiviral activity of IgG fractions. Graph shows the EC_50_ results of high and moderate responders against Delta variants, including IgG data obtained from sera of day-150 post 1st vaccine dose.

As for Delta variant, both IgG groups from day-28 sera showed good anti-SARS-CoV-2 activity, EC_50_ were 10.3 μg/ml for IgG^high^s and 31.8 μg/ml for IgG^moderate^s, and maintained relatively favorable EC_50_ values for IgGs from day-90 sera, EC_50_ were 22.0 μg/ml for IgG^high^s and 65.9 μg/ml for IgG^moderate^s (Fig. 5, Table 4B). This favorable antiviral activity of IgG^high^s against Delta variant was maintained at least 150 days post 1st dose (Fig. 5, Table 4B).

Against Kappa variant, IgG from day-28 sera relatively maintained anti-SARS-CoV-2 activity with EC_50_ values of 20.1 μg/ml for IgG^high^s and 51.0 μg/ml for IgG^moderate^s, and EC_50_ values elevated up to 62.1 and 124.1 μg/ml for day-90 IgG^high^s and IgG^moderate^s, respectively (Fig. 4C, Table 4A). As for E484K variant, similar results were observed with those of Delta variant (Fig. 4C, Table 4A).

## Discussion

In our previous report, we examined neutralizing activity and spike S1-binding antibody (IgG and IgM) response in 225 healthy individuals [physicians (n = 36), nurses (n = 125), and other healthcare professionals (n = 64)], who received two doses of 30 µg BNT162b2 (Pfizer/BioNTech with 3-week interval between 1st and 2nd doses) vaccine in February 2021 in Japan, and investigated the correlation among neutralizing activity levels, S1-binding-IgG and -IgM levels, genders, and adverse events, and time-dependent decline of BNT162b2-elicited immune response^24^. In the present study, we focused on the blood samples collected at 60 days post 1st dose of BNT162b2 vaccination and determined neutralization activity represented by 50% neutralization titers (NT_50_; fold) of each serum and antiviral activity represented by 50% effective concentrations (EC_50_; μg/ml) of each purified IgG fraction by employing the inhibition of virus-induced cytopathic effect employing the target VeroE6^TMPRSS2^ cells and infectious SARS-CoV-2s. Among infectious SARS-CoV-2 strains we used, a Beta variant, containing K417N, E484K, N501Y, and D614G mutations in the spike-encoding region, showed the most significantly reduced susceptibility profile with % reductions of average NT_50_ being 88.5 and 83.4% for high and moderate responders, respectively, compared to those against SARS-CoV-2^05-2N^. Liu et al*.* previously reported that plaque reduction neutralization assay employing blood samples from 15 participants on 2–4 weeks post 2nd dose of BNT162b2 and recombinant virus that contained all the spike mutations of B.1.351 (Beta), showed 63.5% reduction in 50% plaque reduction neutralization titer (PRNT_50_) compared to that against a parental USA-WA1/2020 strain^25^. Our results in the present report are consistent with the data reported by Liu et al. Although there is a difference in the degree of reduction of neutralization activity against Beta variant between Liu’s report and ours, it’s interpretable because there are some differences between their and our experiments, such as assay methods, origins of virus used (recombinant virus or clinically isolated virus), numbers of selected participants, and the timing of blood collection.

The basic design of the present prospective study was (i) to screen the entire cohort (n = 225) in a middle-sized hospital in Japan, where 225 healthcare professionals voluntarily participated in the current study for determining the neutralizing activity of their sera/IgG preparations using the infectious SARS-CoV-2 (the ancestral Wuhan strain)-employed, cell-based assay system; and (ii) to examine the sera/IgG preparations’ neutralizing activity against various SARS-CoV-2 variants of randomly selected high (n = 15) and moderate (n = 30) responders from the same cohort. The scheme was that as many as 45 randomly selected participants’ sera/IgG preparations will produce a set of data, which represents the nature of immunologic features of the entire cohort. Thus, throughout the present study, the sera/IgG preparations from the 45 participants were examined for their neutralizing activity against various variants of concerns (VOCs; Alpha, Beta, Gamma, and Delta variants) and a variant under monitoring (VUM; Kappa variant).

When the decline of neutralization activity is compared on day-28, -60, -90, and -150 post-1st dose, neutralization of high responders’ sera tends to be slow and the activity remained effective longer than that of moderate responders’ sera. This is perhaps due to the MOI effects in the cell-based analysis where if the blockade of viral infectivity and replication is not less, more daughter virions would be produced, thus increasing greater MOI effects than when the blockade is more potent. It is assumed that similar events should occur in the in vivo situation where if the antiviral effect is not potent enough in tissues of the infected individuals, more daughter virions are produced and more pathogenic events are to follow.

The temporal order of the emergence of variants based on the earliest dates of documented samples is: SARS-CoV-2^05-2N^ (the ancestral Wuhan strain; China December 2019), SARS-CoV-2^TY8-612^ (Beta; South Africa May 2020), SARS-CoV-2^QHN001^, SARS-CoV-2^QK002^ and SARS-CoV-2^QHN002^ (Alpha; UK September 2020), SARS-CoV-2^K1734^ (Delta; India October 2020), SARS-CoV-2^K5356^ (Kappa; India December 2020), and SARS-CoV-2^TY7-501^ (Gamma; Japan/Brazil January 2021) (https://www.who.int/en/activities/tracking-SARS-CoV-2-variants/). However, the susceptibility of such variants to neutralization activity of the sera/IgG preparations did not get less in that temporal order. Notably, among the variants examined in the present study, the Beta variant (TY8-612) was least susceptible to the neutralizing activity of IgG preparations from both high and moderate responders, while the Alpha and Delta variants, which appeared later than the Beta variant, were more susceptible to neutralizing activity than the Beta variant (Figs. 2, 3). These data probably suggest a co-expansion nature of each SARS-CoV-2 variant among neighboring and even distant countries and regions with diverse sources and through diverse transmission routes. Moreover, the chronological order of the predominant emergence of the variants is influenced by the transmissibility and pathogenicity acquired by each variant. Thus, it is not surprising that the order of susceptibility of variants to neutralization of sera/IgG preparations from the vaccinated individuals did not match the temporal order of the emergence of the variants examined in the current study.

**Figure 6:**
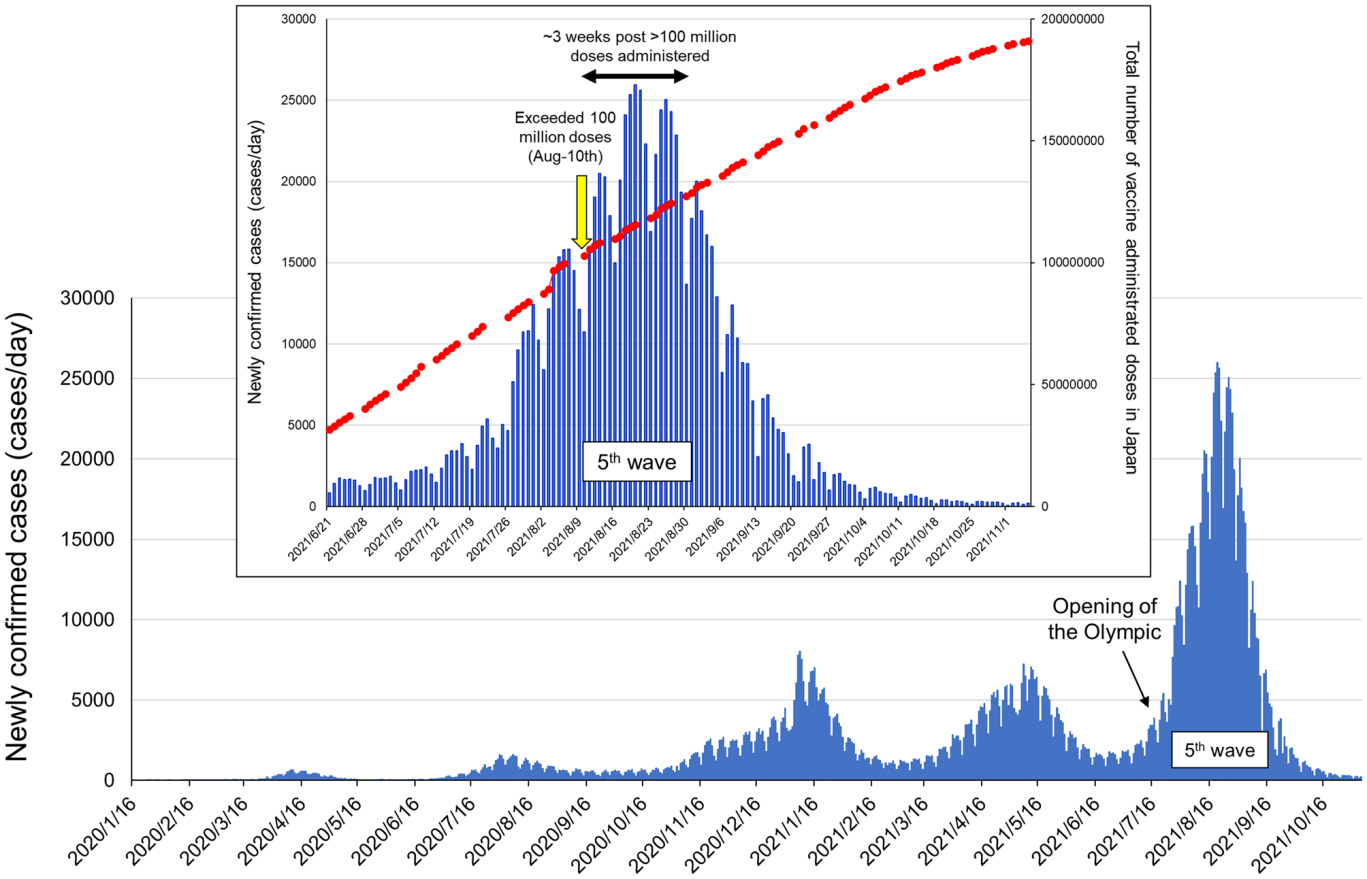
SARS-CoV-2 pandemic in Japan. Newly confirmed SARS-CoV-2-positive cases per day in Japan from early 2020 to Nov-2021 are shown in blue bars. The 5th wave occurred from July-2021 till Oct-2021 in Japan is illustrated in an enlarged view, and total number of vaccine administrated doses in Japan are shown in red.

When we examined and analyzed the time-dependent reduction (day-28 to -90 post 1st vaccine dose) in IgG’s antiviral activity against all the SARS-CoV-2s used in the present study (Figs. 4, 5, Table 4), two variants originated from India (Delta and Kappa variants) showed distinguish profiles against neutralizing activity elicited by BNT162b2 vaccination. The Kappa variant showed moderate to high immune evasion profile probably due to characteristic E484Q mutation in the spike-encoding region, but mutation in this amino-acid does not exist in the Delta variant. It was interesting finding in the present study that purified IgG from sera obtained from 2 doses of BNT162b2 vaccinated Japanese individuals showed relatively long-lasting (at least 150 days post 1st vaccine dose) favorable neutralizing activity against the Delta variant (Fig. 5, Table 4). In Japan, the most severe SARS-CoV-2 pandemic (5th wave) arose from end of July-2021^26^ (Fig. 6), concurrently with an opening of the Olympic games, and % of COVID-19 by Delta variant infection-confirmed cases reached ~ 90% of 5th wave in early-August. The Japanese government has been accelerating SARS-CoV-2 vaccination recently (since mid-June 2021), number of vaccine administrated doses in Japan reached over 100 million doses on August-10th, and since 3 weeks later, number of newly confirmed SARS-CoV-2 positive cases began to decline rapidly afterwards^26,27^ (Fig. 6). These data and our results suggest that the successful antecedent scale-up of mRNA-based vaccination in Japan since June-2021 probably was the primary contributor to the moderation of the otherwise devastating SARS-CoV-2 pandemic, which was caused by the Delta variant susceptible to BNT162b2-elicited immunity. Indeed, the SARS-CoV-2 vaccines used in Japan by Aug-10th, 2021 were mRNA-based vaccines only; BNT162b2 (Pfizer/BioNTech) and mRNA-1273 (Moderna). In addition, the percentages of total vaccine doses of BNT162b2 and mRNA-1273 used in Japan by Aug-10th, 2021 were 81% and 19%, respectively. Thus, as for the possibility that natural-infection-acquired-immunity had moderated the 5th wave, the percentage of total SARS-CoV-2-infected cases in Japan at the beginning of the pandemic (Jan-22nd, 2020 to Aug-10th, 2021) was less than 1% of the entire Japanese population (0.83%); hence, it’s unlikely that natural-infection-acquired-immunity contributed to the moderation of the 5th wave of SARS-CoV-2 infection in Japan.

The present data that the effectiveness of vaccine against the then-dominant SARS-CoV-2 variant was likely associated with the moderation of the COVID-19 pandemic wave suggest that as in the case of influenza vaccines^28,29^, the development of multivalent (multi-SARS-CoV-2-spike-antigen in the case of COVID-19) mRNA-based vaccines represent a generalizable approach to pre-emptively respond pandemic with mutable pathogens.

## Materials and methods

### Serum specimens

Serum samples were collected from 225 vaccinated health care workers at JCHO Kumamoto General Hospital (Kumamoto, Japan). Samples were analyzed at Kumamoto University in Kumamoto and the National Center for Global Health and Medicine (NCGM) in Tokyo. The Ethics Committee from the Kumamoto General Hospital and NCGM approved this study (Kumamoto General Hospital No. 180, and NCGM-G-004176-00). Each participant provided a written informed consent, and this study abided by the Declaration of Helsinki principles. The vaccination (on days 0 and 21, 30 μg/each dose) and serum collection (from day-7, -28, -60, -90 and -150 post 1st dose; number of participants on day-7, -28, -60, -90, and -150 were 225, 220, 211, 210, and 208, respectively) were conducted and carried out.

### Cells and viruses

VeroE6^TMPRSS2^ cells^30^ were obtained from Japanese Collection of Research Bioresources (JCRB) Cell Bank (Osaka, Japan). VeroE6^TMPRSS2^ cells were maintained in DMEM supplemented with 10% FCS, 100 µg/ml of penicillin, 100 µg/ml of streptomycin, and 1 mg/ml of G418. SARS-CoV-2 NCGM-05-2N strain (SARS-CoV-2^05-2N^) was isolated from nasopharyngeal swabs of a patient with COVID-19, who was admitted to the NCGM hospital^31,32^. Nine clinically isolated SARS-CoV-2 variants were used in the current study. hCoV-19/Japan/QHN001/2020 (SARS-CoV-2^QHN001^, GISAID Accession ID; EPI_ISL_804007), hCoV-19/Japan/QK002/2020 (SARS-CoV-2^QK002^, GISAID Accession ID; EPI_ISL_768526), hCoV-19/Japan/QHN002/2020 (SARS-CoV-2^QHN002^, GISAID Accession ID; EPI_ISL_804008), hCoV-19/Japan/TY7-501/2021 (SARS-CoV-2^TY7-501^, GISAID Accession ID; EPI_ISL_833366), and hCoV-19/Japan/TY8-612-P0/2021 (SARS-CoV-2^TY8-612^, GISAID Accession ID; EPI_ISL_1122890) were provided from National Institute of Infectious Disease, Japan. hCoV-19/Japan/TKYTK5356/2021 (SARS-CoV-2^5356^, GISAID Accession ID; EPI_ISL_2378733), hCoV-19/Japan/TKYK01734/2021 (SARS-CoV-2^1734^, GISAID Accession ID; EPI_ISL_2080609), hCoV-19/Japan/TKY76107/2021 (SARS-CoV-2^76107^, GISAID Accession ID; EPI_ISL_1041946), and hCoV-19/Japan/TKY76137/2021 (SARS-CoV-2^76137^, GISAID Accession ID; EPI_ISL_1041947) were provided from Tokyo Metropolitan Institute of public Health, Japan. Each variant was confirmed to contain each VOC or VUM-specific amino acid substitutions.

### Neutralization assay

The neutralizing activity of sera from vaccinated individuals was determined by quantifying the serum-mediated viral suppression in SARS-CoV-2-infected VeroE6^TMPRSS2^ cells as previously described with minor modification^31^. In brief, each serum was serially diluted in culture medium. The diluted sera were incubated with 100TCID_50_ of viruses at 37 °C for 20 min (final serum dilution were 1:20, 1:62.5, 1:250, 1:600, 1;1000 and 1:2000), after which the serum-virus mixtures were inoculated to VeroE6^TMPRSS2^ cells (1.0 × 10^4^/well) in 96-well plates. SARS-CoV-2 strains used in this assay are as follows: a wild-type strain, 05–2N, three Alpha strains, QHN001, QK002, and QHN002 (these variants contain N501Y/D614G mutations in spike), a Beta strain TY-8 (K417N/E484K/N501Y/D614G in spike), a Gamma strain TY-7 (K417T/E484K/N501Y/D614G in spike), a Delta strain TKYK01734 (L452R/T478K/D614G in spike), a Kappa strain TKYTK5336 (L452R/E484Q/D614G in spike),, and two E484K/D614G mutations carrying variants, SARS-CoV-2^F-76107^ and SARS-CoV-2^F-76137^. After culturing the cells for 3 days, the levels of virally caused cytopathic effect (CPE) observed in SARS-CoV-2-exposed cells were determined using the WST-8 assay employing Cell Counting Kit-8 (Dojindo, Kumamoto, Japan). The serum dilution that gave 50% inhibition of CPE was defined as the 50% neutralization titer (NT_50_). Each serum was tested in duplicates.

### Isolation of IgG fractions from vaccinated individual’s sera

Serum samples were collected from vaccinated participants, and IgG fractions were purifed using a spin column-based antibody purifcation kit (Cosmo Bio, Tokyo, Japan) according to the manufacturer’s instructions. In brief, serum was collected, heat-inactivated for 30 min at 56 °C, and spin columns were centrifuged at 3500 rpm for 5 min. IgG fractions in supernatants were eluted and collected. IgG concentration of each obtained fraction was determined by using NanoVue Plus (GE healthcare Japan, Tokyo).

### Anti-SARS-CoV-2 activity assay of eluted IgG

Anti-SARS-CoV-2 activity of eluted IgG fractions were assessed by determining EC_50_ (50% effective concentration) value which inhibit 50% CPE induced by SARS-CoV-2-infection, using serially diluted IgG (final IgG concentration were 2, 10, 50, 100, 200, and 300 mg/ml) and the WST-8 assay, the details of WST-8 assay are described in the “Neutralization assay” section.

### Statistical analyses

We compared 50% neutralization titer values of day-60 sera between SARS-CoV-2^05-2N^ and each variant using the Wilcoxon signed rank test in the high and moderate responders, respectively. Similarly, we compared 50% effective concentration values of day-60 sera using the paired t-test. We assessed the mean of fold changes at day-60, day-90, and day-150 against day-28 with respect to each virus strain and tested the difference between the values of high and moderate responders using Student’s t-test. We calculated all results with R, version 4.1.2 (R Foundation for Statistical Computing).

## Supplementary Information

Supplementary Information.

## Data Availability

The datasets analyzed during the current study available from the corresponding author on reasonable request.
