# Supplementary material for: Neutralization activity of sera/IgG preparations from fully BNT162b2 vaccinated individuals against SARS-CoV-2 Alpha, Beta, Gamma, Delta, and Kappa variants
Source: Sci Rep. 2022 Aug 8;12:13524. doi: 10.1038/s41598-022-17071-9 (PMC9358380; doi:10.1038/s41598-022-17071-9)
Supplement: Supplementary file 1 — Supplementary Information. [file 41598_2022_17071_MOESM1_ESM.pptx]

## Slide 1
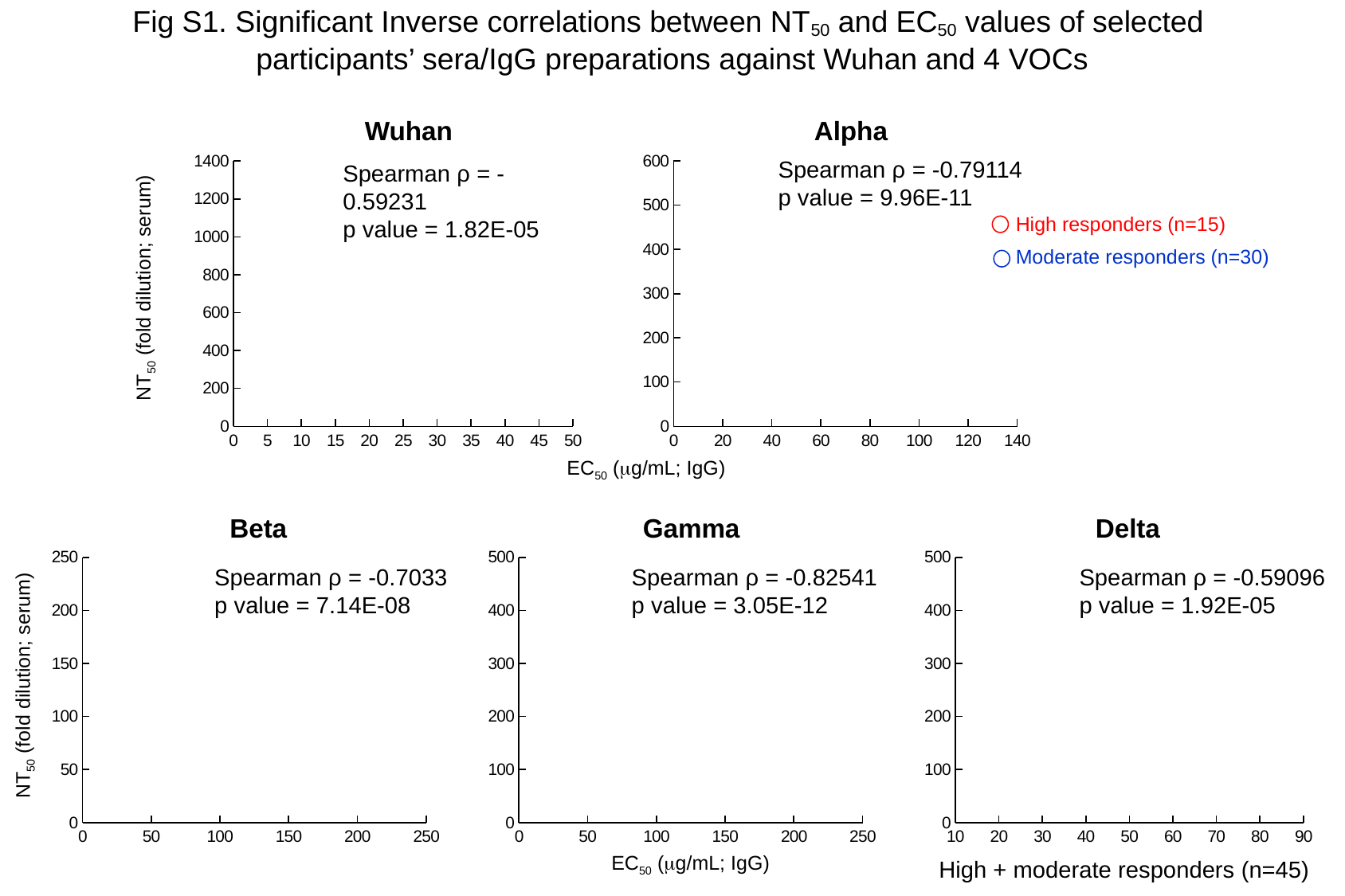

Fig S1. Significant Inverse correlations between NT50 and EC50 values of selected
participants’ sera/IgG preparations against Wuhan and 4 VOCs
Wuhan
Alpha
### Chart
| Category | |
|---|---|
### Chart
| Category | |
|---|---|Spearman ρ = -0.79114
p value = 9.96E-11
Spearman ρ = -0.59231
p value = 1.82E-05
High responders (n=15)
Moderate responders (n=30)
NT50 (fold dilution; serum)
EC50 (mg/mL; IgG)
Beta
Gamma
Delta
### Chart
| Category | |
|---|---|
### Chart
| Category | |
|---|---|
### Chart
| Category | |
|---|---|Spearman ρ = -0.7033
p value = 7.14E-08
Spearman ρ = -0.82541
p value = 3.05E-12
Spearman ρ = -0.59096
p value = 1.92E-05
NT50 (fold dilution; serum)
EC50 (mg/mL; IgG)
High + moderate responders (n=45)

## Slide 2
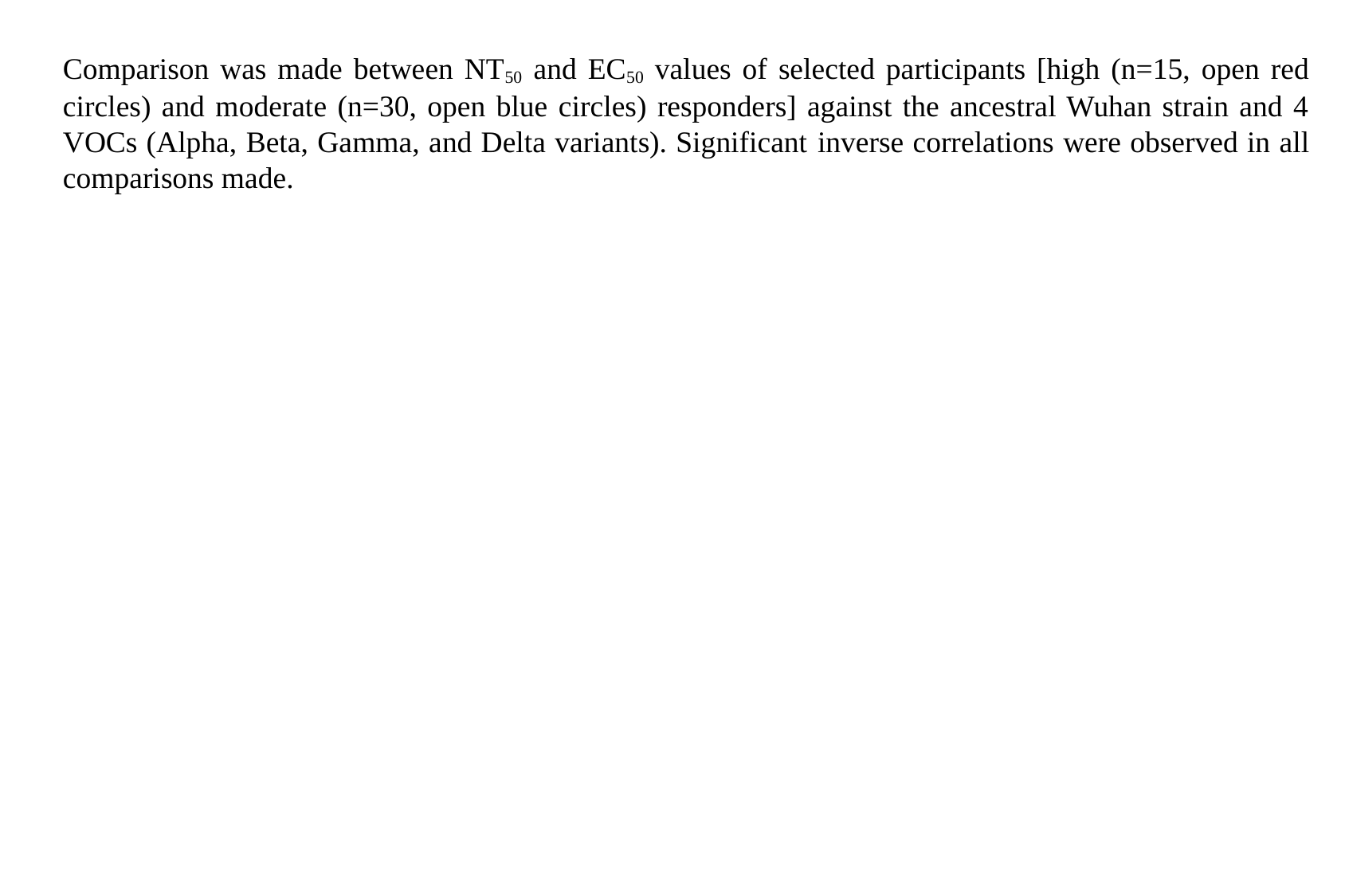

Comparison was made between NT50 and EC50 values of selected participants [high (n=15, open red circles) and moderate (n=30, open blue circles) responders] against the ancestral Wuhan strain and 4 VOCs (Alpha, Beta, Gamma, and Delta variants). Significant inverse correlations were observed in all comparisons made.
